# Physiological, Behavioral, and Life-History Adaptations to Environmental Fluctuations in the Edible Dormouse

**DOI:** 10.3389/fphys.2020.00423

**Published:** 2020-05-05

**Authors:** Thomas Ruf, Claudia Bieber

**Affiliations:** Research Institute of Wildlife Ecology, Department of Interdisciplinary Life Sciences, University of Veterinary Medicine, Vienna, Austria

**Keywords:** pulsed resources, tree masting, seasonal adaptation, hibernation, torpor, huddling, reproduction skipping

## Abstract

The edible dormouse (*Glis glis*, formerly *Myoxus glis*) is a small arboreal mammal inhabiting deciduous forests in Europe. This rodent shows behavioral and physiological adaptations to three types of environmental fluctuations: (i) predictable seasonal variation in climate and food resources (ii) unpredictable year-to-year fluctuation in seed-production by trees and (iii) day-to-day variation in ambient temperature and precipitation. They cope with seasonally fluctuating conditions by seasonal fattening and hibernation. Dormice have adjusted to tree-mast fluctuations, i.e., pulsed resources, by sensing future seed availability in spring, and restricting reproduction to years with at least some seed production by beech and oak trees, which are a crucial food-resource for fast-growing juveniles in fall. Finally, dormice respond to short-term drops in ambient temperature by increased use of daily torpor as well as by huddling in groups of up to 24 conspecifics. These responses to environmental fluctuations strongly interact with each other: Dormice are much more prone to using daily torpor and huddling in non-reproductive years, because active gonads can counteract torpor and energy requirements for reproduction may prevent the sharing of food resources associated with huddling. Accordingly, foraging activity in fall is much more intense in reproductive mast years. Also, depending on their energy reserves, dormice may retreat to underground burrows in the summers of non-reproductive years, causing an extension of the hibernation season to up to 11.4 months. In addition to these interactions, responses to environmental fluctuations are modulated by the progression of life-history stages. With increasing age and diminishing chances of future reproduction, females reproduce with increasing frequency even under suboptimal environmental conditions. Simultaneously, older dormice shorten the hibernation season and phase-advance the emergence from hibernation in spring, apparently to occupy good breeding territories early, despite increased predation risk above ground. All of the above adaptions, i.e., huddling, torpor, hibernation, and reproduction skipping do not merely optimize energy-budgets but also help to balance individual predation risk against reproductive success, which adds another layer of complexity to the ability to make flexible adjustments in this species.

## Introduction

Constant environments are rare. Consequently, animals commonly have to cope with environmental fluctuations. These fluctuations are predictable if they originate from celestial motions of the earth, such as yearly and daily cycles in solar radiation. In many cases, however, variation of the environment, such as fluctuations in local food availability or short-term weather conditions, can be largely unpredictable. Natural selection has therefore favored rheostasis, i.e., the ability of animals to respond to environmental fluctuations by repeatedly changing and readjusting regulated physiological and behavioral levels (Mrosovsky, 1990). These adjustments can affect both energy intake, e.g., foraging behavior or digestive function, and energy expenditure, e.g., the regulation of metabolic rate, body temperature or locomotor activity.

Among mammals, challenging environmental conditions will have a particularly strong impact on small species. This is because of high rates of heat loss, due to a high body surface to volume ratio, which render small endotherms highly susceptible to cold load. At the same time, their high energy demands mean that small mammals can quickly suffer from shortages of food (e.g., Bronson, 1987). A prime example of small mammal exposed to multiple environmental fluctuations is the edible dormouse, a ∼100 g mammal with an arboreal lifestyle that inhabits deciduous forests in western and central Europe. *Glis glis* is an ancient mammal and belongs to the *Gliridae*, which diverged from the *Sciuroidea* at the transition between Paleocene and Eocene, ∼55 MYA, at the time of general rodent diversification (Huchon et al., 2002). The rise of and diversification of the *Gliridae* was likely enhanced by the warm climate in Eurasia that supported the spread of glirid-favored deciduous forests (Nunome et al., 2007). Subsequently, overall earth surface temperatures have considerably declined (Zachos et al., 2001). Simultaneously, edible dormice have acquired a number of rheostatic responses to flexibly adjust their energy turnover in response to environmental challenges. These responses include adaptive modifications in physiology, morphology, and behavior. Below, we examine major responses of this species, namely adaptations to regular seasonal environmental cycles, to unpredictable pulsed food resources, and to rapid fluctuations in weather conditions.

## Seasonal Adaptation

“Stuffed dormouse. Is stuffed with a forcemeat of pork and small pieces of dormouse meat trimmings, all pounded with pepper, nuts, laser, broth. Put the dormouse thus stuffed in an earthen casserole, roast it in the oven, or boil it in the stock pot” (Vehling, 2012). This recipe in the collection “*De re coquinaria* (On the Subject of Cooking),” which is thought to have been compiled in the first century AD, nicely illustrates the origin of the common name “edible dormouse” for *G. glis*. Dormice were probably considered a delicacy in the Imperial Rome because this hibernator can accumulate particularly large amounts of fat. The ancient Romans fattened dormice in earthen jars (*Dolia*) and seemed even to compete for the heaviest dormouse at banquets (Carpaneto and Cristaldi, 1995). For instance, in central Europe (Austria), free-living adult dormice emerge in spring (April/May) with a body mass of 91.1 ± 1.2 g, and reach 128.1 ± 1.1 g prior to hibernation (September/October; Ruf and Bieber, unpublished, *n* = 953, data from 13 years). Some individuals reach a body mass of >200 g prior to hibernation in the field (Löhrl, 1963, Ruf and Bieber, unpublished) as well as in captivity (Bieber and Ruf, 2009b). On average, there is a 40% gain of body mass over the summer season, which appears to be entirely due to the deposition of white adipose tissue (Schaefer et al., 1976). This puts body fat reserves in dormice in the same range (30–50%) as in phocid seals at parturition, which are well known to use extremely large maternal lipid reserves for lactation at sites far removed from food resources (Oftedal, 2000).

Given the allometry of energy expenditure, a small mammal storing large amounts of body reserves is well adapted only if their use is associated with a profound reduction of metabolic rate. Consequently dormice, like many other small mammals, enter states of torpor during hibernation in which energy expenditure is temporarily lowered to only ∼5% of basal metabolic rate (Ruf and Geiser, 2015). Immediate energy savings during the torpid state will be even higher because they allow the animals to avoid maintaining large body-to-ambient temperature gradients during winter. During hibernation, dormice abandon their nocturnal, arboreal activity, cease foraging, and retreat to underground caves or self-dug hibernacula, mostly solitary but occasionally communally in groups of 2–3 (Jurczyszyn, 2007; Kryštufek, 2010; Trout et al., 2018). Thus, hibernation is the most conspicuous behavioral response of dormice to seasonal cold and shortness of food.

Hibernation duration in *G. glis* is relatively long and variable (7–9 months) (Vietinghoff-Riesch, 1960; Kryštufek, 2010; Bieber et al., 2018). Part of this variability is due to the occurrence of reproduction, which delays the onset of hibernation among adults of both sexes (Bieber et al., 2018). Variation in hibernation patterns is also caused by differences in ambient temperature (*T*_a_). First, the duration of torpor bouts that are periodically interrupted by arousals strongly depends on *T*_a_, in *G. glis* as well as in other hibernators (French, 1982; Buck and Barnes, 2000; Bieber and Ruf, 2009b). As the frequency of arousals increases with *T*_a_, this accelerates the depletion of energy stores, since warming up from torpor is costly (Wang, 1979; Strijkstra, 1999; Nowack et al., 2019). Ultimately, this energetic impact of warm temperatures, which does affect the animals in relatively shallow (∼30–80 cm) burrows (Jurczyszyn, 2007; Bieber and Ruf, 2009b; Hoelzl et al., 2015) may force the animals to terminate hibernation early. Second, the seasonal cycle of hibernation as well as of body mass or gonadal function may involve an endogenous rhythm, although this rhythm under experimental conditions had a period of ∼6 months only (Scott and Fisher, 1976; Jallageas et al., 1989). The synchronizing zeitgeber for this rhythm apparently is not photoperiod, however, like in most other hibernators, but seems to be the yearly cycle in *T*_a_, which is why edible dormice have been called thermoperiodic (Jallageas et al., 1989). Both effects of *T*_a_ may cause flexible adjustment of the pattern and duration of hibernation to climatic conditions. Presently, however, it remains to be seen whether the duration of the hibernation season differs as systematically with geographical distribution and climate as seems to be the case in the closely related garden dormouse (Giroud et al., 2014).

A third, and quite different, cause of variation in the use, pattern, and duration of hibernation are trade-offs linked to the use of torpor. Drawbacks of torpor are reflected by mammals avoiding or minimizing this strategy if external or body energy reserves permit (Humphries et al., 2003; Bieber et al., 2014; Zervanos et al., 2014). Dormice with high body mass, for instance, minimize torpor bout duration and regulate higher minimum *T*_b_ during hibernation. Negative consequences of torpor may include sleep deprivation, impaired immune function and oxidative stress (e.g., Daan et al., 1991; Deboer and Tobler, 1994; Carey et al., 2000; Prendergast et al., 2002; Blagojevic et al., 2011). In dormice, oxidative stress seems to be particularly associated with rewarming from deep *T*_b_ which leads to the shortening of telomeres, the protective endcaps of chromosomes (Hoelzl et al., 2016a; Nowack et al., 2019). Telomeres can be elongated, but this is energetically costly, which creates a powerful trade-off between the costs and benefits of hibernation at low *T*_b_ (Hoelzl et al., 2016a; Nowack et al., 2019).

Energy savings are, however, not the only benefit of hibernation, this way of overwintering is also a predator-avoidance strategy. Free living edible dormice reach a maximum longevity of 14 years, which is long for a small rodent (Trout et al., 2015). Longevity is, however, quite typical for small hibernators (Turbill et al., 2011). It may seem peculiar that it should pay for small rodents to allocate resources to somatic maintenance to increase longevity, given that they typically suffer from high extrinsic mortality, namely via predation (Kirkwood, 1977). As it turns out, however, hibernators have much higher survival rates during winter than during the active season, and hence low overall extrinsic mortality (Turbill et al., 2011). Decreased winter-mortality is also typical for edible dormice, and this pattern occurs in different populations across Europe (Lebl et al., 2011a). Predator avoidance is presumably due to torpid animals remaining largely motion- and odorless in hibernacula, which makes them difficult to detect (Turbill et al., 2011; Ruf et al., 2012). This ability of deeply torpid mammals to evade predators has even been demonstrated experimentally (Brown, 1970).

To avoid predators, hibernators should remain hidden in hibernacula at times when predator activity is highest. In dormice, this is in early spring and early summer when their main predators, especially owls, are raising their young and exhibit peak foraging activity (e.g., Forsman et al., 1984). Mortality rates in edible dormice as well as in the closely related common dormice (*Muscardinus avellanarius*) are in fact highest at this time of the year (Lebl et al., 2011a; Bieber et al., 2012). This temporal pattern creates another trade-off since hibernators generally may benefit from early emergence in spring, because it allows individuals to occupy good territories and gain additional mating opportunities (Davis and Murie, 1985; Morbey and Ydenberg, 2001). Hence, early termination of hibernation can increase reproductive success in squirrels, marmots, and hamsters (Murie and Harris, 1982; Millesi et al., 1999; Franceschini-Zink and Millesi, 2008; Ozgul et al., 2010) and prolongs the time for pre-hibernation fattening.

**FIGURE 1:**
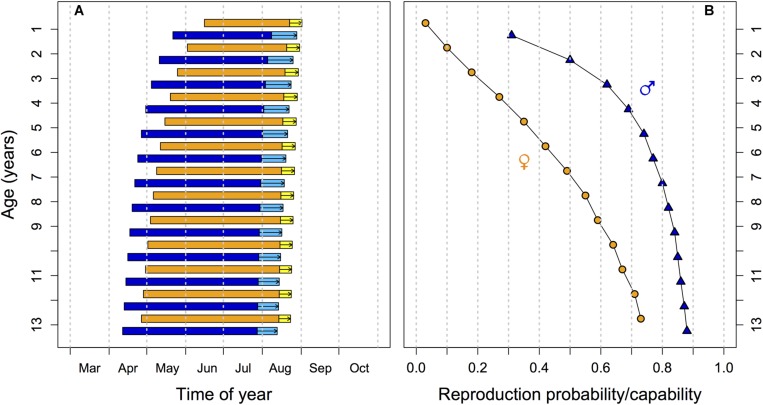
Effects of age on **(A)** the timing of hibernation and activity and **(B)** on reproduction in yearlings and adult edible dormice. **(A)** As age increases both emergence from hibernation and hibernation onset occur earlier in the year, leading to a phase advancement of the active season (horizontal bars; blue: males; orange: females). Investment into reproduction leads to a delay in hibernation onset in both sexes (light blue and yellow bars with arrows). **(B)** The proportion of sexually active males with large testes (blue triangles) and of reproducing females (orange circles) increases with age. Predictions from multilevel models are based on data in Bieber et al. (2018).

The timing of the hibernation season, particularly the time of spring emergence should therefore result from conflicting needs for safety versus above-ground activity and reproduction. As it turns out, the outcome of this trade-off is age-dependent (Figure 1). With increasing age, edible dormice phase-advance the entire hibernation season, with a most pronounced forward-shift of spring emergence. Simultaneously, there is an increase of reproductive capability (functional testes) in males and the probability of having a litter (in females) with progressing age, both in outdoor enclosures and by free-living animals (Hoelzl et al., 2016b; Bieber et al., 2018). We attribute the increased likelihood of reproductive activity in older animals to increasing mortality and diminishing chances for future reproduction (Bieber et al., 2018), which are not only related to age *per se* but also to high year-to-year variation in food resources (see below). In other words, old dormice cannot afford to ‘sit tight’ until environmental conditions are optimal for reproduction. Thus, hibernation is not just altered by variation in energy stores or rates of energy expenditure, as outlined above. The interaction between age, reproduction and the timing of torpid states in dormice indicates that hibernation is also a life history trait, the use and timing of which is adjustable given an animals age and residual reproductive value.

The timing of emergence from hibernation in spring is also sex-specific (Vietinghoff-Riesch, 1960; Bieber, 1998; Schlund et al., 2002). In edible dormice, as in many hibernators, males emerge before females (Michener, 1983; Körtner and Geiser, 1998; Blumstein, 2009; Lane et al., 2011). Early emergence of males is thought to promote individual reproductive success by maximizing the number of potential mates available. Also, males have to spend several weeks at normothermic *T*_b_ after hibernation to complete spermatogenesis (Christian et al., 1972; Barnes et al., 1986).

The hibernation season is, however, merely one of several stages in a seasonal cycle in dormice. Other, equally important facets of this cycle include seasonal growth and shrinkage of alimentary organs such as the liver, which, along with the up- and downregulation of foraging activity contribute to the seasonal cycle of body energy reserves (Bieber et al., 2011, 2017). Also, dormice do not only reproduce seasonally but undergo gonadal involution during winter (Jallageas et al., 1992), which will further add to energy savings. Since all of these responses in mammals are interconnected, in fact they have been called an adaptive syndrome (Heldmaier and Lynch, 1986), it appears that any variation in seasonality is not only due to environmental factors but also affected by the state of animals within their life cycles.

## Pulsed Resources

**FIGURE 2:**
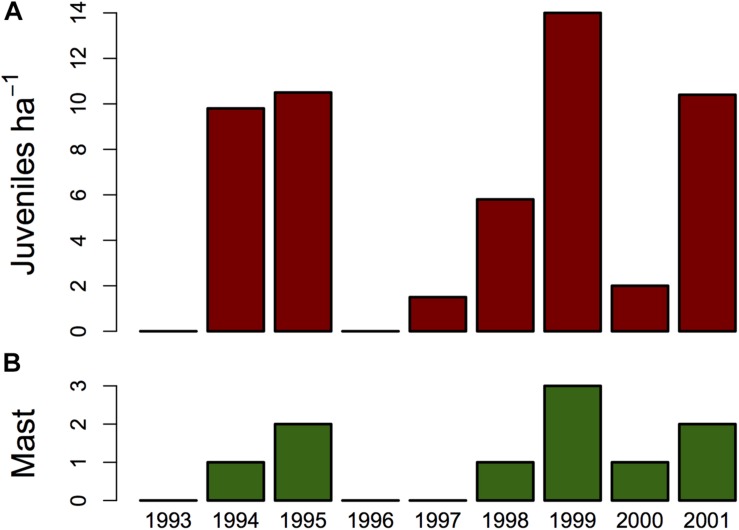
**(A)** Year-to-year fluctuations in edible dormouse reproduction and density as determined by nest box controls over nine years at two study sites (data combined) in southwest Germany. Reproduction was low or even absent in 1993, 1996, 1997, and 2000. **(B)** This was correlated with a low or absent mast of beech trees. Bars represent the degree of beechnut production (codes 0–3 mean absence of beechnuts, some, good, and full mast, respectively). A rank correlation between this index and the number of juveniles detected per year was highly significant (Spearman’s rho 0.965, *n* = 9, *P* = 0.007). Based on data in Ruf et al. (2006).

Edible dormice do not just feed on tree seeds, but also on foliage, flowers, pollen, fruits, berries, lichens, mushrooms, arthropods, and birds, which suffice to gain mass in non-reproductive years (Santini, 1978; Bieber, 1998; Koppmann-Rumpf et al., 2003; Fietz et al., 2005; Vekhnik, 2019). In fact, dormice populations can persist at sites with a very low seed-tree abundance, although with a lower lifetime reproductive output (Bieber and Ruf, 2009). Dormice do, however, respond strongly to year-to-year fluctuations in the mast seeding of deciduous trees, particularly beech and oak, by coupling reproduction to the availability of high-caloric seeds (Bieber, 1998; Schlund et al., 2002; Pilastro et al., 2003; Fietz et al., 2005; Ruf et al., 2006; Vekhnik, 2019). The fraction of successfully reproducing females is high in full mast years with virtually all trees seeding, average in intermediate mast years, when only a fraction of trees produce seeds, and entire populations of dormice can skip reproduction in mast failure years (see Figure 2; e.g., Fietz et al., 2005; Ruf et al., 2006).

The evolution of mast seeding of trees may have resulted from increasing wind pollination efficiency or from satiation of seed predators, such as mice, wild boar, or insects (Silvertown, 1980; Kelly et al., 2001). Regardless of the underlying reason, there are no easily detectable prior signs of masting in the subsequent year, which makes them an example of unpredictable pulsed resources (Ostfeld and Keesing, 2000). Despite this, edible dormice are adapted to these pulses, at least in beech forests, apparently by anticipating the availability of energy-rich seeds at the time of lactation in August through the consumption of unripe beechnuts just after emergence from hibernation in May (Bieber, 1998; Fietz et al., 2005). This explanation has been reinforced by a supplementary feeding experiment, in which the provision of high caloric seeds prior to parturition greatly increased the proportion of reproducing females and reproductively active males in an Austrian population (Lebl et al., 2010).

Thus, the presence of flowers or unripe seeds appears to be the signal that induces reproduction in dormice. Males respond to nutrient-rich food, even prior to the emergence of females, by the growth of testes (e.g., Bieber, 1998; Lebl et al., 2010). Whether the consumption of seed buds also triggers gonadal recrudescence in females is presently unclear, but in populations at the center of the distribution range there are no signs of sexual activity or pregnancy in females, irrespective of their body mass, in years of reproduction skipping (Bieber, 1998; Schlund et al., 2002). In this context, we tested the hypothesis that, in mast failure years, females may be forced to spend longer times foraging for low-quality food, which would increase their exposure to predators. Theoretically, this may lead to chronic stress, i.e., long-term increased secretion of glucocorticoids, which can have inhibitory effects on reproductive function in both female and male mammals (e.g., Boonstra, 2013). However, it turns out that females actually forage more intensively in full mast years (Bieber et al., 2017) and accordingly have higher stress-hormone levels in reproductive years (Cornils et al., 2018). Hence, stress hormones are not the proximate factor linking reproduction to beech seeding in dormice.

Certain aspects of the patterns observed in central/western European beech forests differ from the periphery of the distribution range, e.g., Russia. These peripheral populations also display extreme year-to-year fluctuations in reproduction, in this case linked to the seeding of oak (*Quercus robur*). However, in oak-dominated forests up to ∼90% of males develop functional testes and most females gestate even in years of minimal reproductive output, but there is a synchronous mass resorption of embryos (Vekhnik, 2019). Vekhnik (2019) suggested that time constraints during the short active period in these habitats forces mating to occur rapidly after hibernation, when the degree of fruit or seed set of oak is uncertain, which is why the ‘reproductive decision’ must be made during gestation.

**FIGURE 3:**
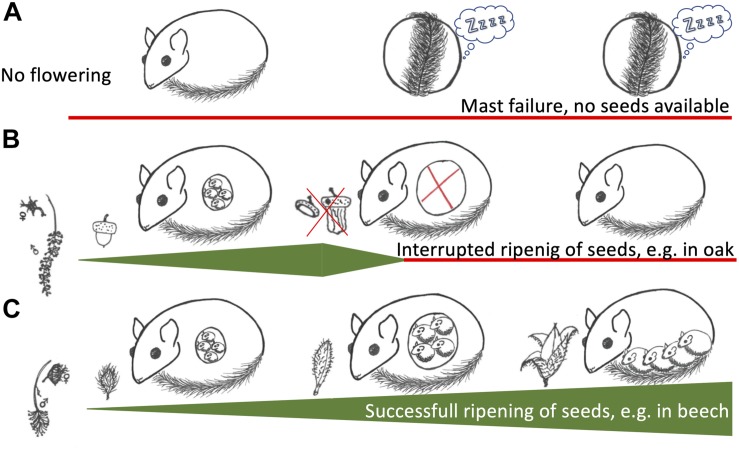
Schematic depiction of reproductive decisions in edible dormice. **(A)** A situation where trees like beech or oak are not flowering, a mast failure year. In these years, reproduction will be skipped and aestivation and prolonged hibernation is observed. **(B)** In certain tree species, such as oak (*Quercus spec*.), there is no correlation between flowering and seeding. Under these circumstances, females may be gestating if seed buds are available but resorb embryos if the food availability decreases. **(C)** In beech (*Fagus sylvatica*) there is a strong correlation between flowering and actual seeding. Under these conditions, females experience a steadily increasing food availability, which allows high food availability for juveniles to fatten after birth.

We suggest that the key to understanding different reproductive patterns of edible dormouse populations is in fact the degree of uncertainty about seed production. In beech, there is a clear correlation between flowering (as indicated by pollen abundance) and subsequent seed production. In contrast, in oaks, pollen abundance is not a predictor of acorn production. The amount of actual oak seed production is strongly affected by weather conditions (Kasprzyk et al., 2014; Bogdziewicz et al., 2017; Lebourgeois et al., 2018). We hypothesize that dormice in both beech and oak forests, actually under all environmental conditions, use the same simple tactic to regulate reproductive investments (Figure 3). In this scenario, individuals of both sexes will, upon emergence from hibernation, initiate investment into gonadal function and reproduction only if food resources with a high energy and nutrient content, e.g., inflorescences or seed buds, are abundant. Females will maintain this investment into reproduction only if high-quality food continues to be present, otherwise reproduction is aborted by embryo resorption. Due to the high reliability of beech, this tactic would lead to a population-wide reproduction versus reproduction skipping in beech masting and mast failure years, respectively. In oak-dominated forests it would lead to high rates of embryo resorption, whenever environmental conditions hamper the production and ripening of oak seeds, as observed in the Russian population (Vekhnik, 2019). Cases of embryo-resorption probably also occur in beech forests, particularly in intermediate mast years, but may well go unnoticed if they occur in early gestation. It would be highly interesting to investigate this question more closely by using imaging methods. Rather than absorbing embryos, occasionally, females may eat their pups after parturition if food is not available. This would explain observations of entire litters vanishing in an extremely hot summer in which beech nuts were present but empty, i.e., when even beech trees were an unreliable food source (unpublished). Of course, basing reproductive decisions on the continuous “sampling” of environmental resource availability may also be adaptive in other habitats inhabited by dormice, such as pine forests or stands with fruit-bearing trees (Santini, 1978; Bieber and Ruf, 2009a).

In the beech forests of their central distribution the observed reproductive pattern (Figure 2) seems like an example of anticipatory reproduction, in which reproductive decisions are made before a resource pulse, i.e., ripe seeds, becomes available. An apparent prediction of future food abundance has been reported for a few other small mammals (e.g., Berger et al., 1981; Boutin et al., 2006; Bergeron et al., 2011). However, the existence of anticipatory reproduction has been disputed by White (2007, 2013), who pointed out that females require substantial amounts of protein to produce embryos, sustain their growth, and support the amount and quality of lactation. In the scenario outline above, this condition is met because the unripe seeds eaten by females, like all reproductive parts of plants, likely contain sufficient amino acid sources to initiate reproduction (Fietz et al., 2005; White, 2011). In addition to its role as a nutrient source, high-quality food may, however, also serve as an environmental signal that predicts the advent of ripe, energy-rich beechnuts in August–September, coincident with lactation. In this context, it would be interesting to assess whether dormice are particularly sensitive to the action of hormones that are known to serve as links between energy uptake, white adipose tissue and the reproductive system, such as leptin, ghrelin, and orexins (e.g., Kirsz et al., 2017; Williams et al., 2017).

Irrespective of whether or not the tactic employed by edible dormice may be called ‘anticipatory reproduction,’ there is no doubt that it serves to synchronize their annual peak in energy demand with the annual peak in resource availability, a central tenet in ecology (Thomas et al., 2001; Stenseth and Mysterud, 2002). In central Europe, edible dormice have only one litter per year, with a mean litter size of 5.5 young (Kager and Fietz, 2009; Lebl et al., 2010). Mating takes place from mid-June to mid-July; the gestation period lasts 30–32 days, and juvenile dormice are weaned at approximately 6 weeks of age (Koenig, 1960; Vietinghoff-Riesch, 1960). Thus, dormice synchronize lactation, which is distinctly later than in other hibernating mammals, with the availability of ripe seeds (Bieber and Ruf, 2004). Energy availability is limiting for successful lactation in this species. Females in good condition, with high body mass, breed earlier, while females in poor condition delay pregnancy so that lactation occurs when feeding conditions are at their best (Pilastro et al., 1994). Lactation creates extremely high energy demands, and supplemental feeding during the lactation period leads to increased growth rates of young (Zoufal, 2005; Kager and Fietz, 2009). Accordingly, heavy females produce young with a higher mean body mass at weaning, but litter size is not affected by female body mass (Pilastro et al., 1994; Kager and Fietz, 2009).

The high costs of lactation mean that, among mammals, females frequently invest more into reproduction than males (e.g., Clutton-Brock, 1991). Thus, given the relatively low energetic cost of maintaining functional testes, it seems puzzling that in dormice males forego any chances for reproduction by remaining entirely sexually inactive. In mast-failure years most males maintain regressed testes, even when some older females do reproduce, due to little chances to survive to the next year (Schlund et al., 2002; Ruf et al., 2006; Hoelzl et al., 2016b). One reason for this unusual behavior may be the fact that in mast failure years juveniles sired by reproductively active males are probably unable to acquire sufficient fat reserves prior to hibernation, which leads to negligible chances of winter survival. In addition, a long-term capture-recapture study has shown that reproduction in dormice is costly in terms of survival in both males and females, leading to the counter-intuitive pattern that survival is higher in poor years of low food abundance (Ruf et al., 2006). This effect of reproduction was found in different populations across Europe, and the negative impact of reproduction was even slightly greater in males than in females (Lebl et al., 2011a). Reduced survival probably originates mainly from the greater foraging activity and associated predation risk in reproductive years (Bieber et al., 2017; Cornils et al., 2018) but may also be caused by energetic bottlenecks. As pointed out by Fietz et al. (2004, 2005) male dormice pay a much higher energetic price for sexual competence than that caused by spermatogenesis alone. This is because testosterone production by functional testes prevents males from using short bouts of torpor during the summer-active season (Jallageas and Assenmacher, 1983). This pathway of energy saving is therefore restricted to non-reproductive years (Fietz et al., 2004, 2010; Bieber et al., 2017). In the early summer of reproductive years, males actually lose body mass, likely due to high costs of competition for mates (Bieber, 1998; Pilastro et al., 2003; Ściński and Borowski, 2008). Thus, in years without beechnuts as a high-quality food resource, males might simply not be able to cope with these costs and fail to develop functional testes (Fietz et al., 2005).

The regulation of reproduction in dormouse is, however, far from just an on/off switch linked to mast seeding. Particularly interesting, in this context, are intermediate years in which only a fraction of females have litters. A detailed study on reproductive decisions under these circumstances (Lebl et al., 2011b) confirmed that adult animals are more likely to have offspring in any particular year than yearlings (see also Figure 1), but that body mass is not a crucial factor determining whether a female will reproduce, even in an intermediate year (Bieber, 1998; Pilastro et al., 2003; Fietz et al., 2005). This independence of reproductive investment from energy stores makes dormice typical income breeders (Stephens et al., 2014; Williams et al., 2017). A decisive environmental factor affecting reproduction turned out to be the age of the beech forest in the home ranges of females. The proportion of females reproducing strongly increased with the mean age of trees, reaching close to 100% at a tree age of ≥80 years (Lebl et al., 2011b). Arguably, this is because older trees produce more seeds, and they also produce seeds more frequently (Nilsson and Wästljung, 1987; Yamauchi, 1996; Genet et al., 2009). Somewhat surprisingly, a high proportion of beech compared with other tree species had either no impact on reproduction (in most years), or even a negative effect on the number of females reproducing during a year with low-intermediate beechnut availability. This suggested that, in years with little seed production, areas with low beech density may provide better resources for reproduction, because there are often alternative foods such as fruits, berries, or other seeds (Lebl et al., 2011b). As mentioned before, dormice populations may persist and reproduce at low rates even in the absence of large seed trees (Bieber and Ruf, 2009a). In view of these facts it may be less surprising that an environmental niche factor analysis revealed that dormice actually avoid areas with high beech tree density, but instead prefer areas with a relatively high proportion of coniferous trees (Cornils et al., 2017). Conifer cones and leaves are another alternative food source for edible dormice (e.g., Santini, 1978). Coniferous trees also have fluctuating mast seeding events, but in the distribution range of dormice there are often several conifer species, which show only moderate synchrony of seeding (Koenig and Knops, 1998). Consequently, their collective seed production is much more stable over time than that of beech (Cornils et al., 2017). Accordingly, areas dominated by conifers can sustain the same densities of dormice as in beech forests, and dormice in more diverse mixed beech and conifer forests have even higher survival rates (but lower reproductive output) than dormice in beech forests (Schlund et al., 2002; Ruf et al., 2006).

Local resource availability for the investment into reproduction is, however, only one of the factors that determines habitat suitability for dormice. While coniferous forests interspersed with mature beech may be optimal in terms of reproductive success, there also other aspects that determine habitats, particularly predation risk. Edible dormice most frequently inhabit forests with closed canopies and stands with mid-canopies and high vertical stratification (Cornils et al., 2017), features that are avoided by their main predators, aerial-hunting owls (Hunter et al., 1995). Hence, it appears that suitable habitats are mainly characterized by predator avoidance, because vertical stratification of forests means a lower mean age of trees and hence suboptimal resource availability (Lebl et al., 2011b; Cornils et al., 2017). Since dormice are able to escape from predation almost completely by retreating to underground burrows, it seems logical that they should do so even in summer in years of reproduction skipping, when there is no good reason to stay active. As it turns out this is exactly what dormice do, at least the fraction of individuals that can afford to do so energetically.

**FIGURE 4:**
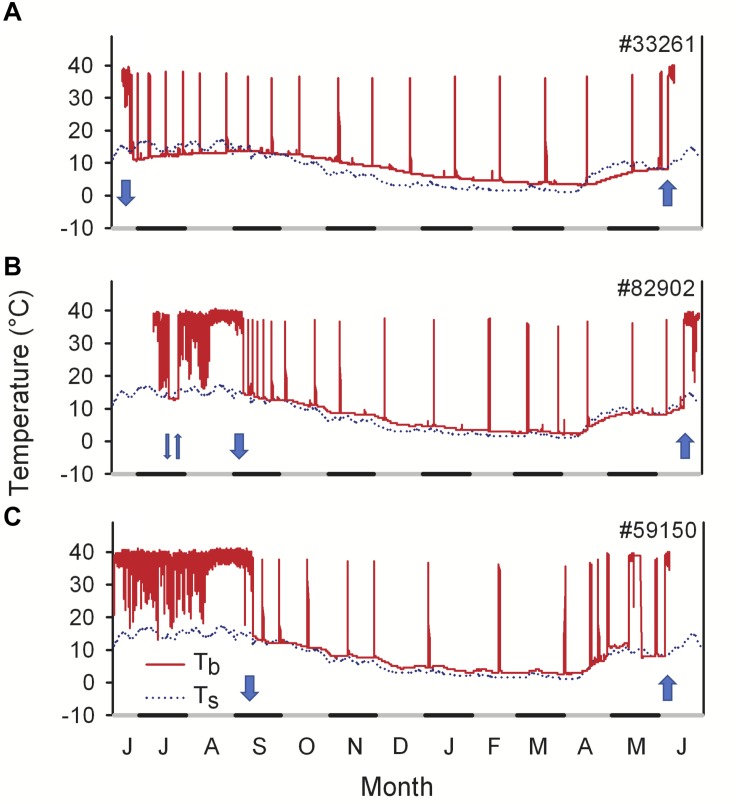
Year-round records of core body temperature (*T*_b_, solid lines) in three free-living edible dormice in 2012/2013. **(A)** Example of an animal showing an early onset of hibernation in June and staying in hibernation for 331 days. **(B)** An animal using multiday torpor during the active season in July. **(C)** An individual showing a long active season, entering hibernation in September. Torpor *T*_b_ during hibernation and summer dormancy was close to soil temperature (*T*_s_, dotted lines). The arrows indicate retreat into (arrow down) and emergence from (arrow up) underground hibernacula. Modified from Hoelzl et al. (2015).

Dormice with a high body mass at emergence can, after an active period of only a few weeks, retreat to hibernacula again in June (Hoelzl et al., 2015). They immediately commence hibernation (or technically “estivation,” during the summer) until the next spring, for a total duration of up to 11.4 months (Figure 4). Thus, the animals briefly emerge after an 8 months hibernation period only to enter hibernation for almost another year, resulting in 19 out of 21 months spent hibernating (Bieber and Ruf, 2009b). Because dormice are fasting during hibernation, this behavior requires extremely large fat stores, and was observed only in approximately 50% of the animals. Thus, the onset of estivation occurred progressively earlier as body mass increased (Hoelzl et al., 2015). However, dormice estivate as deep as >60 cm below ground (Hoelzl et al., 2015), i.e., at relatively low *T*_a_ during summer, which limits arousal frequency und thus energy expenditure.

A hibernation duration of more than 11 months, which is longer than in any other free-living mammal, may be the most effective means of predator avoidance (Turbill et al., 2011; Geiser and Brigham, 2012). Importantly, estivation is not induced by energetic constraints in spring (Bieber and Ruf, 2009b) but appears to be an example of anticipation of the lack of future resources for successful reproduction. In other words, dormice respond to pulsed resources by making use of equally pulsed periods of extensive hypometabolism and cryptic behavior. This adaptation seems to occur in various populations. For instance, in an 18-year study in England, Trout et al. (2015, 2018) found that of those animals known to be alive, ca. 90% were captured in nest boxes in reproductive years but only 10–35% in non-reproductive years, presumably because the unseen animals were estivating. In fact, dormice routinely appear to vanish during summer, as indicated by low recapture rates (of living animals) in non-reproductive years in different populations across Europe (Lebl et al., 2011a).

## Rapid Fluctuations

Even if a substantial fraction of animals are dormant in non-reproductive years, the individuals remaining active, and almost all dormice in reproductive years, will be exposed short-term changes in external conditions. These rapid changes, particularly due to weather, are the third type of environmental fluctuations that may induce physiological and behavioral adjustments. Among rodents, responses of activity levels, thermoregulation, or social behavior to weather conditions are common (e.g., Kenagy, 1973; Vogt and Lynch, 1982; Gilbert et al., 2010; Williams et al., 2014b; Wróbel and Bogdziewicz, 2015).

Currently, there are no detailed studies of activity patterns by edible dormice under changing weather conditions. However, in the closely related common dormouse (*M. avellanarius*) activity is strongly influenced from night to night, mainly by *T*_a_. Lower *T*_a_ has the effect of shortening activity, and cold nights may promote diurnal activity on rare occasions. There is also a weaker negative impact of rainfall on activity length in spring (Bright et al., 1996). Bright et al. (1996) suggested that hibernators are able to greatly reduce foraging activity because they can compensate for the ensuing reduced energy intake by facultative torpor.

This would also apply to the edible dormouse, who may not only exhibit hibernation and estivation but also short bouts of torpor (<24 h, Figures 4B,C). These bouts are often called daily torpor (e.g., Wilz and Heldmaier, 2000) although it is not clear if these short bouts in hibernators are physiologically equivalent to those in daily heterotherms. Metabolic rates during short bouts of torpor are close to hibernation metabolic rates and a magnitude lower than typically recorded during daily torpor in daily heterotherms (Wilz and Heldmaier, 2000; Ruf and Geiser, 2015). This contributes to large energy savings via short bouts of torpor, which can reach up to 80%, depending on its duration (3–21 h; Wilz and Heldmaier, 2000). In free-living dormice, short torpor is largely restricted to the photophase and on average lasts ∼7.5 h (Hoelzl et al., 2015). Torpor bouts mostly occur early in the active season, predominantly on cool days (Figures 4B,C; Hoelzl et al., 2015; Bieber et al., 2017). However, during cold spells in spring or early summer, dormice can also prolong hypometabolism and undergo multiday bouts of torpor, i.e., estivate (Figure 4B). Minimum *T*_b_ during multiday torpor frequently falls well below *T*_a_, indicating that these episodes occur almost certainly in underground burrows (Hoelzl et al., 2015).

As mentioned before, the use of torpor seems to be incompatible with reproduction in dormice (Fietz et al., 2010), although not in all mammals (McAllan and Geiser, 2014). Consequently, short bouts of torpor occur almost exclusively in non-reproductive years and only rarely in mast years, prior to the reproductive season (Bieber et al., 2017). There may be a link in the physiological regulation of torpor and locomotor activity, because in dormice there is a direct correlation between frequent torpor bouts and low activity, and the animals are much more active in reproductive years (Bieber et al., 2017). It may seem that this association merely reflects the fact that dormice may use torpor to compensate for low foraging duration and hence reduced food intake, induced by low *T*_a_ (Bright et al., 1996). However, daily heterotherms may have low rates of activity during periods of high torpor use even when food is abundant (Ruf et al., 1991; Ruf and Heldmaier, 2000). Arguably, this is because frequent torpor lowers total daily energy requirements, which in turn lowers the need for foraging. It is this complementary reinforcement which explains that energy savings via torpor and the associated reduction in activity are much higher than to be expected from hypometabolism in torpor alone (Ruf et al., 1991; Ruf and Heldmaier, 2000).

In dormice, any reduction in locomotor activity will significantly reduce energy expenditure, even independent of torpor use. In mast seeding years, dormice cease the use of short torpor with the onset of reproduction, and are highly active during the late summer season. While foraging at night under these conditions, both sexes reach core *T*_b_s exceeding 40°C for several hours (Bieber et al., 2017). This indication of high foraging effort is most pronounced in lactating females, and is typical of the expensive lifestyle of arboreal mammals (Karasov, 1981). Any reduction of these costs of activity, as in non-reproductive years (Bieber et al., 2017), should result in profound energy savings.

**FIGURE 5:**
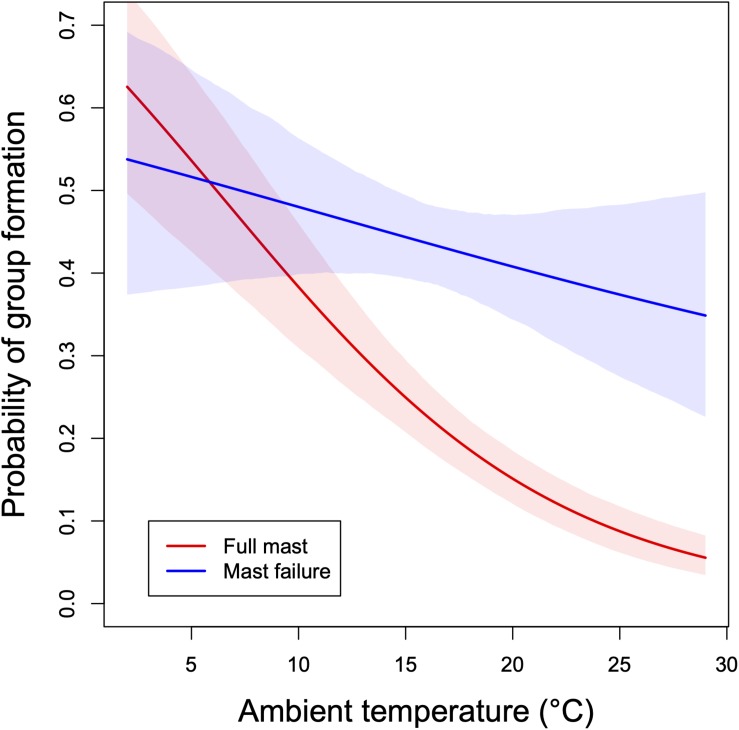
Probability of group formation as a function of ambient temperature during the past 24 h in edible dormice. Groups consisted of 2–16 adults and yearlings of both sexes. Predictions ± 95% credible intervals from a Bayesian multilevel model for years of full mast seeding (red) and mast failure years (blue) of beech trees. Data from a 13-year mark-recapture study, Austria. Recomputed from data in Ruf and Bieber (2020).

Short torpor, brief episodes of estivation, and adjustments of activity are not the only responses of edible dormice to fluctuating environmental conditions. It has long been known that dormice inhabiting nest boxes (as a substitute for natural tree holes), while often solitary, aggregate in groups of up to 24 adults in a single box (Gatter and Schütt, 1999). We recently found that these groups, in that study consisting of up to 16 dormice, are aggregating predominantly in response to cold in the previous night (Figure 5; Ruf and Bieber, 2020). Also, these short-term aggregations during the daily resting phase are mainly formed by smaller animals, often yearlings, which have higher rates of heat loss due to a high surface to volume ratio (Fietz et al., 2010; Ruf and Bieber, 2020). Thus, the principal function of this group formation is social thermoregulation, i.e., huddling (review in Gilbert et al., 2010).

Not unexpectedly, the proclivity for huddling in dormice varies between mast seeding and mast failure years. In mast seeding/reproductive years, both sexes appear reluctant to aggregate and form huddles only at low *T*_a_ (Figure 5). However, their capability to huddle even in reproductive years indicates that elevated levels of reproductive hormones do not prevent this behavior. Aggregations during the daily resting phase come at the cost of having to share food resources during foraging at night. Indeed, at least in beech forests home-ranges in both sexes are relatively small, except for lactating females (ca. 0.8 ha, Hönel, 1991). Apparently, the high energy demands of competition for mates and investment into reproduction select against sharing of food resources in reproductive years. Lactating females in particular never seem to share nest boxes with males, although they can occasionally communally nest with related females (Pilastro, 1992; Ruf and Bieber, 2020). In contrast, in mast-failure years the tendency for huddling is higher, even at warmer *T*_a_s (Figure 5).

Dormice mitigate the consequences of resource competition by preferentially huddling with relatives. This is achieved by ‘family reunions’ of mothers and siblings, or siblings only, which join the same groups as yearlings and adults, suggesting that this behavior has evolved through kin selection (Ruf and Bieber, 2020). Costs of resource competition also appear to be alleviated by a surplus of males, which probably have lower energy demands than reproducing females (Ruf and Bieber, 2020). Remarkably, despite the potential of the aggression-promoting effects of testosterone, aggregations include males with fully developed testes (Fietz et al., 2004, 2010; Ruf and Bieber, 2020). As pointed out by Fietz and colleagues, for sexually active males that do not use torpor, social thermoregulation may be the only response to cold left (Fietz et al., 2010). Interestingly, the use of huddling, predominantly in mast failure years, means that dormice show strong year-to-year shifts in their degree of sociality. These alterations seem rare but have been also reported in the sister-clade of the dormice, the Sciurids, in which females of otherwise ‘asocial’ species may communally nest outside the reproductive season (Williams et al., 2013). Of course, social thermoregulation and other tactics to adverse environmental conditions are by no means mutually exclusive. For instance, dormice may use short bouts of torpor while resting in huddling groups (Ruf and Bieber, 2020), and these behavioral responses can facilitate each other, which increases their energetic efficiency (Ruf et al., 1991; Jefimow et al., 2011; Eto et al., 2014).

## Synopsis: Comparative Aspects

The primary responses of edible dormice to seasonal changes in food availability and climate, namely, seasonal reproduction followed by energy storage, gonadal involution and hibernation are common among mammals. At least 93 species of mammals from 11 orders are known to hibernate (Ruf and Geiser, 2015) and the cessation of energy-demanding reproductive processes during the hibernation season is the rule (but see Morrow and Nicol, 2009). However, the extent of hibernation- use in dormice is extremely large. This concerns both their average (8 months) and maximum hibernation duration (>11 months), which is significantly longer than, for instance, among the Marmotini (Williams et al., 2014a). Arguably, these characteristics are mainly due to the high energy density of seeds [7.0–7.9 kcal/g dry mass (dm); Grodziński and Sawicka-Kapusta, 1970], the preferred food of dormice in reproductive years. It is this high-caloric food which enables rapid fattening as well as extremely fast growth of young, factors that allow for the production of single litter very late in summer, compared with other hibernators (Bieber and Ruf, 2004). In those hibernators predominantly feeding on herbage during the reproductive season [4.0–4.6 kcal/g dm; (Hutton, 1962)], such as several ground squirrels and marmots, juvenile growth is much slower. This slow development favors reproduction early in the season and thus constrains the hibernation duration.

The second source of environmental variation affecting dormice, mast-seeding of trees with large changes in energy availability, creates strong pulses in reproductive output. This response is not uncommon, however. Other seed predators such as American and Eurasian tree squirrels (*Tamiasciurus hudsonicus*) also anticipate resource pulses and increase reproductive output before a masting event, whereas another squirrel (*Sciurus vulgaris*) increases reproduction following mast seeding of Norway spruce (Selonen et al., 2015). Responses of dormice most closely resemble those of Eastern chipmunks (*Tamias striatus*) which reproduce intensely prior to American beech mast [*Fagus grandifolia*; (Bergeron et al., 2011)]. Strikingly, just like dormice, chipmunks were found to interrupt aboveground activity for 9–11 months when mast was not available (Munro et al., 2008). However, while Eastern chipmunks are hibernators, this inactivity may not reflect aestivation, because large food hoards enable this species to cease foraging even without the use of torpor (Humphries et al., 2003; Munro et al., 2008).

Pulsed resources do no only affects graminivores. For instance, omnivores, such as the wild boar, have increased reproductive output after mast seeding of beech or oak (Bieber and Ruf, 2005). Similarly, the Kangaroo rat (*Dipodomys merriami*), which requires green plants for reproduction, is a “pulse matcher” that responds directly to the occurrence of pulses of food production (Kenagy and Bartholomew, 1985). Insectivorous bats such- as the lesser mouse-eared bat (*Myotis blythii*) respond to years with superabundance of cockchafers in spring by advancing parturition, and by delaying puberty, compared with their sibling species *Myotis myotis*, which exploits this food resource less (Arlettaz et al., 2017). Insectivores, especially bats, are also the largest group among the mammalian hibernators (45 out of 93 species; Grizmek, 1997; Ruf and Geiser, 2015). Currently it is apparently unknown, however, whether any pulsed resource trackers among these species use extended hibernation during years of low food abundance. Pulsed resources also affect reproduction in insectivorous birds (Ostfeld and Keesing, 2000; Drever et al., 2009), but there are no interactions with hibernation, because there is only one bird species known to hibernate, i.e., to use multiday torpor (Jaeger, 1949). However, many birds use daily torpor in both winter and summer (Geiser, 2010; Ruf and Geiser, 2015), and torpor frequency in birds can be strongly increased by low arthropod abundance (e.g., Doucette et al., 2012). Thus, it seems that a variety of mammals and birds are capable of flexibly using heterothermy to adjust energy expenditure to variations in food availability, far beyond responding only to the typical scarcity of food during winter in temperate and arctic zones. Since this use of heterothermy serves to adjust energy turnover to maximize fitness, it may be viewed as a flexible life-history trait, not just in dormice. Arguably, species adapted to pulsed resources that are synchronized over large geographical zones, such as dormice or chipmunks, are only the most conspicuous examples for these effects, because entire populations respond simultaneously. In other species, effects of reproduction on the timing of hibernation, and its use to adjust energy expenditure to reproductive effort, may occur only locally and/or affect individuals differently (e.g., Michener, 1983; Lehmer et al., 2006; Millesi et al., 2008; Zervanos et al., 2010; Siutz et al., 2016).

The responses of edible dormice to short-term variation in weather conditions, namely short torpor and huddling, are also widespread among endotherms (reviews in Gilbert et al., 2010; Ruf and Geiser, 2015). One interesting avenue of future research in this context would be the joint energetic effects of these responses, in species like dormice that use them simultaneously (cf. Jefimow et al., 2011). Taken together, it seems that edible dormice use a variety of strategies to respond to environmental fluctuations, like hibernation, aestivation, anticipatory reproduction and reproduction skipping, as well as short torpor and huddling, which are not uncommon adaptations among endotherms. The only unusual aspect seems to be their combined occurrence in a single species. One of the most pertinent questions for future research on dormice and other pulsed resource consumers will be the effects of global climate change on mast frequency and the long-term consequences for both trees and seed predators.

## Author Contributions

TR and CB conceived of this review and TR wrote the first draft. CB commented, critically revised, and edited the manuscript. TR and CB read and approved the final version of the manuscript.

## Conflict of Interest

The authors declare that the research was conducted in the absence of any commercial or financial relationships that could be construed as a potential conflict of interest.
